# Response to “Ribosome Rescue and Translation Termination at Non-standard Stop Codons by ICT1 in Mammalian Mitochondria”

**DOI:** 10.1371/journal.pgen.1005227

**Published:** 2015-06-18

**Authors:** Zofia Maria Chrzanowska-Lightowlers, Robert Neil Lightowlers

**Affiliations:** Wellcome Trust Centre for Mitochondrial Research, Newcastle University, Medical School, Newcastle upon Tyne, United Kingdom; Max Planck Institute for Biology of Ageing, GERMANY

## Overview

In a recent paper published by Akabane et al. [1], a homologous mitochondrial translation system was supplemented with ICT1, the mitochondrial translation release factor family member. Under these conditions, ICT1 was shown to exhibit general release activity at all codons tested, including AGA/AGG, which are normally redundant in human mitochondria. The authors suggest that this ICT1 activity may occur in vivo, challenging Temperley et al., 2010 [2]. We wish to point out, however, that the data presented in this paper are in vitro and do not account for the live cell data consistent with a -1 frameshift at AGA/AGG placing a standard UAG stop codon in the human mitoribosomal A-site.

## Response

The impressive data, recently published in *Nature* and *Science*, from the groups headed by Ban and by Ramakrishnan have confirmed, through the use of high-resolution cryo-electron microscopy (cryo-EM) and chemical cross-linking–mass spectroscopy, that ICT1 is a component of the mammalian mitochondria [3–5]. ICT1 is a member of a family of release factors, which are ribosome-dependent peptidyl–transfer ribonucleic acid (tRNA) hydrolases. These proteins all contain a highly conserved tripeptide motif, GGQ, that is essential for promoting cleavage of the ester bond that anchors the nascent peptide chain to the resident tRNA [6–8]. In addition to ICT1, these class I release factor (RF) family members include mtRF1a (mtRF1L), mtRF1, and C12orf65. Only one of these, mtRF1a (mtRF1L), has had a clearly defined physiological function ascribed to it. This protein recognises stop codons UAA and UAG to facilitate release activity [9]. Both ICT1 and C12orf65 are smaller proteins in part due to the loss of the codon recognition domains. Studies to identify their functions, along with those of mtRF1, continue. A little more information is available for ICT1. First principles would suggest that the permanent presence in the ribosome of a protein that can hydrolyse randomly a nascent peptide from the P-site tRNA would be a dangerous design. Experimental evidence has shown that the GGQ motif in ICT1 has retained functionality. Site-directed mutations were introduced in this tripeptide to generate HEK293 cell lines that can express either GSQ or AGQ variants. For each mutant cell line, sucrose gradients and coimmunoprecipitation experiments confirmed incorporation of the variant ICT1 into the mitoribosome. The presence of either version of the mutated ICT1, however, caused growth defects [10]. The fact that mammalian mitoribosomes have adopted this protein as an integral member of the large subunit but do not suffer from profligate abortive translation events indicates that they have also developed a strategy to control this activity.

We were pleased to see the recent article on ICT1 by Akabane et al. that described the use of translationally active mammalian mitoribosomes [1]. This publication presents in vitro data, which show elegantly that purified ICT1 can release di/tripeptides from mammalian mitoribosomes programmed with a variety of short, synthetic, RNA molecules. The authors suggest this result is in disagreement with work previously published from our laboratory. We believe, however, that the data and conclusions reached by Akabane in their recent publication are in general agreement rather than in contradiction to Richter et al. (2010) [10]. In our original paper, we observed that free ICT1, in our in vitro assays, was able to demonstrate translational release activity on bacterial ribosomes lacking messenger RNA (mRNA) or any codon, including AGA and AGG (Fig 3C in Richter et al.) [10]. When ICT1 is in excess and not incorporated into the mitoribosome, it may be able to access the A-site and activate termination of a stalled 55S; it may also be able to do so following slippage of a 55S particle that reached an out-of-frame AGA/AGG or if no mRNA was present in the A-site. The natural state of the cells, however, appears not to retain an excess free pool of ICT1. We performed sucrose gradients to reveal the status of the ribosomal proteins using cells that only contained endogenous levels of ICT1. These show that in HEK293 cells, all detectable protein is integrated into the mitoribosome and that there is negligible, if any, unincorporated ICT1 present (Fig 2E of Richter et al.) [10]. Furthermore, we were careful to say that uncontrolled activity of ICT1 under normal elongation conditions would be predicted to be detrimental because of the potential to abort elongation. In the absence at that time of any structural information on the position of ICT1 in the mitoribosome, we suggested that ICT1 could display rescue activity only under conditions that cause mitoribosome distortion. We are, therefore, not surprised that no release activity was observed under normal conditions in the in vitro reactions of isolated 55S on substrate in the absence of release factors. The lack of activity of mtRF1a/L and mtRF1 on ribosomes lacking any resident transcript was also consistent with our observations. The possibility that ICT1 dissociates under different physiological conditions is an interesting concept that could be readily tested.

Akabane and colleagues employed coupled transcription translation assays in which exogenous ICT1 was added to 70S bacterial ribosomes, an experimental condition close to those of our in vitro assays, which also used 70S supplemented with added release factors. The observed activity of ICT1 is again in agreement with our data in which addition of ICT1 facilitated release activity on paused/stalled ribosomes on all codons tested, including nonstop, UAA, and AGA (Fig 2A of Akabane et al. [1] cf. Fig 3C of Richter et al. [10]).

The use of 70S ribosomes programmed with longer transcripts has allowed more detailed examination of translation processes. Akabane et al. have used this to analyse the action of free ICT1 on ribosomes that have stalled during elongation and found that AGA/AGG can be recognised and release activity invoked [1]. This is consistent with our earlier observations that ICT1 can release P-site amino acids from 70S ribosomes programmed with AGA or AGG, which are paused by virtue of this being the end of the available RNA [10].

The inability to decode AGA/AGG as arginine in mitochondrial translation is well established, but what happens when mitochondrial ribosomes encounter such hungry codons, and are the release factor family members ICT1, C12orf65, or mtRF1 involved? The potential functions of these RF family members, ICT1, C12orf65, and mtRF1, remain unclear. The PXT motif involved in codon recognition has expanded to a hexapeptide in many mammalian mtRF1 proteins. The extension of this motif has been suggested to disrupt the recognition of the first two bases of a termination codon, changing the selectivity from UA to AG, and so it has been proposed that mtRF1 could recognise AGA/AGG [11]. The functions of mtRF family members have more recently been predicted using extensive computer-based molecular dynamic simulations, free-energy calculations, and/or homology modelling to derive 3-D structures in the ribosomal A-site [12,13]. However, these data indicate that neither mtRF1 nor mtRF1a would be able to recognise AGA/AGG in order to facilitate termination at these codons [12,13].

A number of mammalian mitochondrial DNA (mtDNA) sequences have a single AGA or AGG triplet following the *MTCO1*/*MTND6* open reading frames, respectively. These sequences also retain a preceding U that would place UAG in the A-site upon -1 mitoribosomal frameshifting, as was implicated by Temperley et al. [10]. This arrangement, however, is not universal. For example, AGA occurs at the end of the *MTCYB* bovine, ovine, and equine coding sequences in which a directly preceding U is absent. One unifying theme for our work and that of Akabane et al. could therefore be that in mitochondria from these species, other RF members such as ICT1 or C12orf65 promote translational release at the in-frame AGA/G.

The mystery of the interplay between these triplets and the mt-release factor family members is complex. Interestingly, both rat and mouse retain an mtRF1 protein in addition to the termination factor mtRF1a and yet only use UAA to terminate all of the mtDNA encoded open reading frames [14,15]. Moreover, studies with cell lines depleted of these factors or from patients harbouring mutations in *C12orf65* have shown that all four members are important in human mitochondrial translation. It is difficult to be categorical on the functions of these mitochondrial RF family members. Which protein functions on which substrate and under what circumstance remains elusive. For example, it is not unreasonable to extrapolate the in vitro data generated by Akabane et al. [1] to infer that should free ICT1 exist under particular physiological conditions or occur in specific tissues, it could facilitate translation termination at AGA/AGG codons. Crucially, however, the results on stop codon usage by *MTCO1* and *MTND6* as published in *Science* by Temperley et al. [2] were performed in growing cells and reflect the actual stop codon positioned in the A-site [2]. Although the prediction and previous long-term assumption was that AGA/AGG acted as A-site stop codons, this contrasts with the actual derived data in cultured cells, which show this to be UAG.

Whilst we welcome the results of Akabane and colleagues, there remains the enigma of defining the precise role that integrated ICT1 and the two other members of the mitochondrial RF family, mtRF1 and C12orf65, play in mammalian mitochondrial protein synthesis in vivo.
